# Consent to Medical Data Processing Among Adult Primary Care Patients: Associations with Vaccination Behaviour and Healthcare Trust

**DOI:** 10.3390/healthcare14162505

**Published:** 2026-08-12

**Authors:** Agnieszka Rusiecka, Dorota Stefanicka-Wojtas, Aneta Soll-Morka, Tomasz Hikawczuk, Maria Kasprzyk-Smardz, Monika Zadka, Norbert Zachara, Jarosław Zachara, Stanisław Karol Manulik, Joris van Loenhout, Izabella Uchmanowicz, Donata Kurpas

**Affiliations:** 1 Statistical Analysis Centre, Biostatistics Teaching Team, Wroclaw Medical University, 50-368 Wroclaw, Poland; agnieszka.rusiecka@umw.edu.pl; 2 Division of Scientific Research Methodology, Department of Nursing, Faculty of Nursing and Midwifery, Wroclaw Medical University, 51-618 Wroclaw, Poland; dorota.stefanicka-wojtas@umw.edu.pl (D.S.-W.); aneta.soll-morka@umw.edu.pl (A.S.-M.); izabela.uchmanowicz@umw.edu.pl (I.U.); donata.kurpas@umw.edu.pl (D.K.); 3 Medra Kasprzyk-Smardz & Partners, 63-600 Kępno, Poland; medra@kepno.net; 4 Non-Public Healthcare Center “Przychodnia Nowy Dwór”, 54-438 Wroclaw, Poland; monika.zadka@nzoznowydwor.pl; 5 Non-Public Healthcare Center “ReMed”, 32-825 Borzęcin, Poland; norbetr.zachara@gmail.com (N.Z.); jarzach@gmail.com (J.Z.); 6 Division of Healthcare Organization, Department of Nursing, Faculty of Nursing and Midwifery, Wroclaw Medical University, 51-618 Wroclaw, Poland; stanislaw.manulik@umw.edu.pl; 7 Epidemiology of Infectious Diseases, Epidemiology and Public Health, Sciensano, 1050 Brussels, Belgium; joris.vanloenhout@sciensano.be

**Keywords:** medical data processing, informed consent, secondary use of health data, vaccination behaviour, primary care, healthcare trust, preventive healthcare, logistic regression

## Abstract

**Background**: The secondary use of routinely collected medical data is increasingly important for healthcare research and public health. However, the validity and representativeness of consent-based datasets depend on patients’ willingness to allow their medical information to be used. Although vaccination behaviour and trust in healthcare systems have both been extensively studied, little is known about their relationship with consent to medical data processing. **Objective**: This study aimed to identify factors associated with consent to medical data processing among adult primary care patients, with particular emphasis on vaccination status, vaccination attitudes, information-seeking behaviours, and trust-related factors. **Methods**: A cross-sectional study was conducted among 921 adult patients recruited from three primary healthcare centres in Poland representing different organizational and geographical settings. Data were collected using a standardized questionnaire. The primary outcome was participants’ consent to the transfer of vaccination-history data (yes/no), referred to throughout the manuscript as “consent to medical data processing”. Associations between consent status and socio-demographic characteristics, vaccination-related attitudes, information sources, and trust-related factors were assessed using chi-square tests and non-parametric methods. Hierarchical multivariable logistic regression models were constructed to identify factors independently associated with consent. Model performance was evaluated using the area under the receiver operating characteristic curve (AUC), Nagelkerke’s *R*^2^, and the Hosmer-Lemeshow goodness-of-fit test. **Results**: Overall, 565 participants (61.3%) provided consent to medical data processing and 356 (38.7%) declined consent. Among participants who provided consent, 88.5% were classified as vaccinated, compared with 69.1% among those who did not provide consent (*p* < 0.001). In multivariable analyses, vaccination status remained independently associated with consent across all hierarchical models. The fully adjusted model showed that vaccinated participants had more than twice the odds of providing consent compared with unvaccinated individuals (OR = 2.27; 95% CI: 1.32–3.92; *p* = 0.003). Older age was also positively associated with consent (OR = 1.02 per year; 95% CI: 1.01–1.03; *p* = 0.004). Significant and persistent differences were observed between healthcare centres, suggesting that organizational and contextual factors may play an important role. By contrast, vaccination attitudes, trust in the healthcare system, perceived accessibility of vaccination information, and information-source were not independently associated with consent after adjustment. The final model demonstrated good discrimination (AUC = 0.892), satisfactory calibration (Hosmer–Lemeshow *p* = 0.647), and moderate explanatory power (Nagelkerke’s *R*^2^ = 0.557). **Conclusions**: Consent to medical data processing was independently associated with vaccination status and healthcare centre among adult primary care patients. The findings suggest that vaccination behaviour and organizational context are important correlates of consent to medical data processing. Organizational characteristics may also play an important role in shaping consent behaviour in healthcare settings. Researchers using consent-based medical data should consider the potential impact of differential consent behaviour on the representativeness of study populations. Further research is needed to understand contextual and behavioural determinants of consent to medical data processing.

## 1. Introduction

The secondary use of routinely collected medical data has become an increasingly important component of contemporary healthcare research and public health practice [1,2]. Large healthcare databases and patient registries provide valuable opportunities to evaluate healthcare interventions, monitor population health trends, and support evidence-based policymaking. However, the validity and representativeness of such analyses depend critically on patients’ willingness to consent to the use of their medical information [3,4]. When consent is required, individuals who decline participation may differ systematically from those who agree, potentially introducing selection bias and limiting the generalizability of research findings [5,6,7].

Evidence suggests that public acceptance of secondary use of health data is influenced by trust in healthcare institutions, transparency of data governance, perceived societal benefits, and confidence in data protection mechanisms [8,9,10]. Individuals who perceive healthcare organizations as trustworthy are generally more willing to participate in health research and permit the use of their medical records for scientific purposes [11,12,13]. Conversely, concerns regarding privacy, data security, information misuse, or insufficient transparency may reduce willingness to provide consent. Public trust is therefore considered a critical prerequisite for the sustainable use of routinely collected health data in research and healthcare improvement initiatives.

Primary care settings provide a particularly relevant context for studying consent to medical data processing. Primary care physicians often serve as patients’ first and most frequent point of contact with the healthcare system and play a central role in establishing long-term therapeutic relationships. As a result, trust in healthcare institutions, confidence in healthcare professionals, and willingness to engage in health-related activities may be particularly relevant within primary care populations [14]. Furthermore, characteristics of healthcare organizations and local care environments may influence both trust and willingness to participate in healthcare and research activities, including consent to the use of medical data.

Previous research suggests that characteristics of healthcare organizations, including communication practices, patient–provider relationships, continuity of care, and local healthcare environments, may influence patients’ trust, engagement with healthcare services, and participation in health-related activities [13,15,16]. Organizational factors operating at the primary care level have also been associated with differences in patient satisfaction, healthcare utilization, and willingness to engage in preventive health behaviours [15,17]. Together, these findings suggest that the organizational context of primary care may be an important determinant of willingness to consent to medical data processing.

Trust is also a central determinant of preventive health behaviours, particularly vaccination. According to the 5C model of vaccination behaviour, confidence in vaccines, healthcare professionals, and health authorities represents one of the key drivers of vaccine acceptance [18,19,20]. Numerous studies have consistently demonstrated that higher levels of trust in healthcare systems and healthcare providers are associated with greater vaccine uptake and lower vaccine hesitancy [13,21]. Individuals who trust healthcare institutions are more likely to rely on professional sources of information, accept public health recommendations, and engage in preventive healthcare behaviours [22,23,24,25]. In contrast, distrust in healthcare systems has been linked to vaccine hesitancy, increased susceptibility to misinformation, and lower participation in preventive health programmes.

The conceptual overlap between willingness to share medical data and vaccination behaviour suggests that both phenomena may reflect broader patterns of engagement with healthcare systems [3,8]. Individuals who actively participate in preventive healthcare may be more willing to cooperate with healthcare institutions in other domains, such as research activities using medical records. Numerous studies have investigated determinants of vaccine acceptance and public attitudes toward secondary use of health data separately. However, relatively few studies have examined whether consent to medical data processing is associated with vaccination behaviour, vaccination attitudes, and trust-related factors within the same population [23,25,26,27]. Understanding these relationships may provide insights into broader patterns of healthcare engagement and participation in health-related activities.

This issue may be particularly relevant in Central and Eastern European countries, where historical, political, and socio-economic transformations have influenced public attitudes toward state institutions and healthcare systems. Institutional trust remains an important determinant of health-related decision-making in these settings. Consequently, examining factors associated with consent to medical data processing may provide broader insights into trust-related and preventive health behaviours among primary care patients.

Therefore, this study aimed to investigate factors associated with consent to medical data processing among adult primary care patients, with particular emphasis on vaccination status, vaccination-related attitudes, information-seeking behaviours, and trust-related factors. We hypothesized that consent to medical data processing would be associated with vaccination behaviour, attitudinal factors related to vaccination, and trust-related characteristics reflecting engagement with preventive healthcare.

## 2. Materials and Methods

### 2.1. Study Design and Settings

This cross-sectional observational study was conducted in three primary healthcare centres in Poland representing different organizational and geographical settings: a large urban centre, a medium-sized urban centre, and a rural centre. The participating primary healthcare centres were selected through the procurement procedure conducted within the EUVABECO project. Together, they represented diverse primary care settings with respect to patient populations and healthcare delivery. All centres provided primary healthcare services under contracts with the National Health Fund (NFZ). The procedure for obtaining participants’ consent to link questionnaire responses with medical data followed a standardized study protocol across all participating healthcare centres.

### 2.2. Participants

The study was conducted between November 2024 and April 2025. Adult patients attending routine visits at one of the participating primary healthcare centres were recruited consecutively. Eligible patients were informed about the study by healthcare staff and invited to participate voluntarily. After receiving information about the study, participants completed the study questionnaire. Eligible participants were adults (≥18 years) attending one of the participating primary healthcare centres during the study period. Apart from age below 18 years and failure to complete the questionnaire, no predefined exclusion criteria were applied. The final sample of 921 participants therefore comprised all eligible individuals who agreed to participate and completed the study questionnaire during the study period rather than a predetermined target sample size. Participants were recruited from three primary healthcare centres located in different geographical and organizational settings: a large urban centre, a small urban centre, and a rural centre. In addition to geographic differences, these centres differed regarding patient populations, local healthcare environments, and organizational context. Accordingly, the healthcare centres were regarded not only geographically but in terms of their contextual characteristics, reflecting potential differences in healthcare delivery and patient engagement.

Participation involved completion of a questionnaire assessing demographic characteristics, vaccination-related attitudes, trust in healthcare information sources, and perceptions of vaccination policies. Participants provided consent to link their questionnaire responses with selected medical data held by the participating primary healthcare centre for research purposes within the EUVABECO project. The primary outcome of the present study was whether participants provided this consent. Participants who did not provide consent for linkage of vaccination-history data remained eligible for the present analysis because all analyses were based exclusively on questionnaire-derived variables.

### 2.3. Data Collection

Data were collected using a standardized questionnaire developed within the EUVABECO project. The same questionnaire was used across all participating healthcare centres without modification. The questionnaire included items on socio-demographic characteristics, vaccination status, attitudes towards vaccination, trust in healthcare institutions and information sources, sources of vaccination-related information, and perceptions of vaccination policy.

To ensure consistency, identical procedures for participant recruitment, provision of study information, questionnaire administration, and data collection were implemented across all participating healthcare centres. For participants who provided consent for data linkage, questionnaire responses were linked locally within each participating primary healthcare centre. Selected medical data were linked using a unique study identification code. The linkage key remained at the participating healthcare centres and was not accessible to the research team. Only coded (pseudonymized) research datasets were transferred to the research team for analysis. The investigators had no access to information that could enable participant identification.

### 2.4. Variables

All variables analyzed in the present study were obtained from self-administered questionnaires completed by study participants. The questionnaire collected information on socio-demographic characteristics, vaccination status, vaccination-related attitudes, information-seeking behaviours, and trust-related factors. Consent to medical data processing was assessed as a separate questionnaire item and used as the primary outcome variable in this analysis. In the questionnaire, participants were asked whether they agreed to the transfer of their medical data regarding vaccination history (Supplementary Table S1). Responses were coded as 1 (consent provided) and 0 (consent not provided). Throughout the manuscript, the term “consent to medical data processing” is used as an operational label for consistency. It refers exclusively to participants’ consent to the transfer of vaccination-history data within the EUVABECO project, as assessed by the questionnaire item described above, and should not be interpreted as general consent to the secondary use of medical data. No individual-level clinical information from medical records was included in the analyses presented in this manuscript. Socio-demographic variables included age (years), sex (female/male), place of residence (village, city < 50,000 inhabitants, city 50,000–200,000 inhabitants, city > 200,000 inhabitants), and healthcare centre. Additional descriptive characteristics included education, profession, income, and religion.

Vaccination status was recorded as a binary variable (vaccinated/unvaccinated).

In this study of adult participants, classification reflected participants’ current approach to vaccination rather than childhood immunization history. Adults who received at least one vaccination were classified as vaccinated, whereas those who refused all vaccinations were classified as unvaccinated. Participants who accepted some vaccinations but declined others were also classified as vaccinated. Thus, the classification reflected overall current vaccination behaviour and did not refer to any specific vaccine (e.g., COVID-19 vaccination or influenza). A vaccine concerns index was constructed from six questionnaire items addressing concerns about adverse events following immunization, long-term health effects, vaccine composition, potential negative effects on the immune system, the influence of the pharmaceutical industry, and conspiracy-related beliefs regarding population control or microchipping. Responses were measured on five-point Likert scales and summed to create a composite score, with higher values indicating greater vaccine-related concerns. The internal consistency of the six-item scale was good (Cronbach’s α = 0.80), supporting its use of a composite index in subsequent analyses.

Trust-related variables included perceived availability and quality of vaccination-related information, as well as confidence in the healthcare system. These variables were assessed using six-category ordinal scales ranging from “very poor” to “very good”, with an additional “no opinion” category.

Participants also reported whether they used specific sources of vaccination-related information, including physicians, family and friends, internet websites and social media, books and scientific publications, traditional media, governmental organizations, and non-governmental organizations. Trust in these information sources was assessed using five-point Likert scales ranging from “I do not trust at all” to “I fully trust”.

For multivariable modelling, physicians and family/friends were selected as information-source variables based on the previous literature and preliminary analyses. Following the multicollinearity assessment, healthcare centres were retained over place of residence as a variable in the final model because they showed substantial overlap.

### 2.5. Multicollinearity

Prior to model estimation, multicollinearity was assessed using variance inflation factors (VIFs). Variables with VIF values > 5 were considered potentially problematic. Because healthcare centre and place of residence showed substantial overlap, only healthcare centre was retained in the final model. No evidence of problematic multicollinearity was observed in the revised final model (all VIFs < 2.5).

### 2.6. Missing Data

Complete-case analysis was applied. Consequently, the sample size varied across hierarchical models based on data availability for individual variables. Separate complete-case analyses were performed for each hierarchical model; therefore, the analytical sample differed according to the variables included in each model.

### 2.7. Model Performance

Model calibration was assessed using the Hosmer–Lemeshow goodness-of-fit test. Discrimination was evaluated using the area under the receiver operating characteristic curve (AUC). Explanatory power was assessed using Nagelkerke’s *R*^2^.

### 2.8. Statistical Analysis

Statistical analyses were performed using Statistica 13.3 (TIBCO Software Inc., Palo Alto, CA, USA). Continuous variables were summarized using means and standard deviations or medians and interquartile ranges, depending on their distribution. Categorical variables were presented as frequencies and percentages. Associations between categorical variables were assessed using Pearson’s chi-square test. Continuous variables were compared using non-parametric methods when appropriate.

To identify factors independently associated with consent to medical data processing, a series of hierarchical multivariable logistic regression models was developed. This modelling strategy was chosen to determine whether the association between vaccination status and consent remained stable after adjustment for demographic characteristics, vaccination-related attitudes, and trust-related factors. The order of model construction was based on a theoretical framework which assumed that socio-demographic characteristics are distal determinants of health behaviour. By contrast, vaccination status, vaccination attitudes, and trust-related factors were considered progressively more proximal determinants. Place of residence was initially evaluated but was not included in the hierarchical regression models due to substantial overlap with the healthcare centre. The healthcare centre was retained as a contextual variable potentially reflecting differences in local healthcare environments and organizational settings.

Four nested models were estimated.

Model A (socio-demographic model) included age (years), sex (female vs. male), and healthcare centre. Place of residence was initially evaluated but not included in the hierarchical models because of substantial overlap with the healthcare centre.

Model B (vaccination behaviour model) included all variables from Model A as well as vaccination status (vaccinated vs. unvaccinated). This model assessed whether vaccinated individuals were more likely to provide consent independently of socio-demographic characteristics.

Model C (vaccination attitudes model) included all variables from Model B as well as the vaccine concerns index, assessing the influence of others on vaccination decisions (five-point scale), evaluating vaccination policy (five-point scale), and examining attitudes toward mandatory vaccination (yes/no/no opinion). This model examined whether the association between vaccination status and consent is due to differences in vaccination-related attitudes.

Model D (trust and information model) included all variables from Model C in addition to trust in the healthcare system (five-point scale), perceived accessibility of vaccination information (five-point scale), physicians as a source of vaccination information (yes/no), and family and friends as a source of vaccination information (yes/no). This model evaluated whether the association between vaccination status and consent remained significant after controlling for trust-related factors and information-seeking behaviours. Both perceived accessibility and the quality of vaccination information were evaluated in preliminary analyses. As these variables were conceptually related and highly correlated with the outcome, only perceived accessibility was retained in the final multivariable model.

Complete-case analysis (listwise deletion) was applied in all multivariable logistic regression models. The reduction in sample size across successive models resulted from missing responses to questionnaire items introduced at each modelling stage; therefore, different participants were excluded from different models under the complete-case approach. Consequently, comparisons of model performance across models should be interpreted descriptively.

For regression analyses, responses of “no opinion” were treated as missing values and were therefore excluded from the corresponding complete-case analyses.

Ordinal variables were entered into the regression models as continuous predictors, consistent with common practice for Likert-type scales in epidemiological research.

To assess the consistency of the association between vaccination status and consent across healthcare centres, additional centre-specific univariable logistic regression analyses were performed for Healthcare Centres 1 and 2, with vaccination status entered as the sole predictor. Healthcare Centre 3 was excluded in these analyses because only vaccinated participants were represented in the analyzed study sample.

Odds ratios (ORs) with 95% confidence intervals (95% CIs) were reported. Model discrimination was assessed using the area under the receiver operating characteristic curve (AUC), model calibration using the Hosmer–Lemeshow goodness-of-fit test, and explanatory power using Nagelkerke’s *R*^2^. All tests were two-sided, and a *p*-value < 0.05 was considered statistically significant.

## 3. Results

### 3.1. Study Population

A total of 921 adult primary care patients were included in the study. Overall, 565 participants (61.3%) provided consent to the processing of medical data, whereas 356 (38.7%) declined consent.

Vaccination status differed substantially according to consent status. Among participants who provided consent, 88.5% were classified as vaccinated, compared to 69.1% among those who declined consent (*p* < 0.001) (Table 1).

### 3.2. Socio-Demographic Characteristics According to Consent Status

The socio-demographic characteristics of the study participants are presented in Table 2.

Consent rates differed significantly across healthcare centres (*p* < 0.001). Place of residence was also associated with consent status in unadjusted analyses.

No significant differences were observed with respect to sex. Although age was not associated with consent in the unadjusted comparison, a modest positive association emerged after adjustment for contextual factors. Differences in education and religious affiliation were not statistically significant in descriptive analyses.

### 3.3. Vaccination-Related Information and Trust

Perceptions of vaccination-related information and confidence in the healthcare system differed significantly in unadjusted analyses; however, these associations were not retained in multivariable models (Table 3).

### 3.4. Sources of Vaccination Information

Patterns of information seeking related to vaccination also differed according to consent status (Table 4).

Participants who provided consent were substantially more likely to identify physicians as a source of vaccination information than those who declined consent (80.8% vs. 64.9%, χ^2^ = 28.48, *p* = 0.001). Similarly, consenting participants more frequently reported obtaining vaccination-related information from family members and friends (45.1% vs. 33.3%, χ^2^ = 12.17, *p* < 0.001).

No statistically significant differences were observed between groups regarding the use of internet sources, social media and online forums, traditional media (television, radio, and press), books and scientific publications, governmental organizations, or other information sources (all *p* > 0.05).

### 3.5. Trust in Information Sources

Differences in trust in several vaccination-related information sources were examined between participants who consented and those who declined. Detailed results are presented in Supplementary Table S3.

### 3.6. Vaccination Decisions and Attitudes Towards Vaccination Policy

Significant differences in vaccination-related attitudes were observed between participants who consented to medical data processing and those who declined consent (Table 5). Participants who provided consent reported a greater influence of family, friends, and social groups on vaccination decisions (*p* < 0.001), evaluated national vaccination policy more favourably (*p* = 0.009), and were substantially more likely to support mandatory vaccination (61.1% vs. 42.0%; *p* < 0.001). In contrast, participants who declined consent more frequently opposed mandatory vaccination or reported having no opinion on the issue.

### 3.7. Vaccine-Related Concerns

Participants who declined consent to medical data processing reported higher levels of vaccine-related concerns than those who provided consent. These differences were observed across all assessed domains, including vaccine safety, long-term health effects, vaccine composition, perceived effects on the immune system, pharmaceutical industry influence, and conspiracy-related beliefs. Detailed results are presented in Supplementary Table S4.

### 3.8. Hierarchical Logistic Regression Models Predicting Consent to Medical Data Processing

The results of the hierarchical logistic regression analyses are presented in Table 6.

In Model A, which included only socio-demographic and contextual variables, older age was associated with a higher likelihood of providing consent (OR = 1.02 per year; 95% CI: 1.01–1.03; *p* = 0.002). Significant differences were also observed between healthcare centres. Compared with Healthcare Centre 1, participants recruited in Healthcare Centre 2 were substantially less likely to provide consent (OR = 0.07; 95% CI: 0.04–0.10; *p* < 0.001), whereas participants from Healthcare Centre 3 were considerably more likely to provide consent (OR = 7.05; 95% CI: 3.30–15.08; *p* < 0.001). Sex was not significantly associated with consent.

After the inclusion of vaccination status in Model B, vaccinated participants had approximately twice the odds of providing consent compared with unvaccinated individuals (OR = 2.07; 95% CI: 1.33–3.21; *p* = 0.001). The associations observed for age and healthcare centre remained largely unchanged.

Adjustment for vaccination-related attitudes in Model C did not materially alter the relationship between vaccination status and consent (OR = 2.00; 95% CI: 1.23–3.26; *p* = 0.005). None of the attitudinal variables, including the vaccine concerns index, influence of others on vaccination decisions, evaluation of vaccination policy, or attitudes toward mandatory vaccination, were independently associated with consent after adjustment.

In the fully adjusted Model D, vaccination status remained an independent predictor of consent (OR = 2.27; 95% CI: 1.32–3.92; *p* = 0.003), and older age was positively associated with consent (OR = 1.02; 95% CI: 1.01–1.03; *p* = 0.004). The strong association with healthcare centre persisted across all models.

Across all hierarchical models, vaccination status and healthcare centre emerged as the most consistent predictors of consent to medical data processing. Importantly, the association between vaccination status and consent remained stable after adjustment for socio-demographic characteristics, vaccination-related attitudes, trust-related factors, and information-seeking behaviours.

The predictive performance of the hierarchical logistic regression models improved progressively with the sequential inclusion of vaccination status, vaccination-related attitudes, and trust-related variables (Table 7). Model discrimination, assessed using the area under the receiver operating characteristic curve (AUC), increased from 0.872 in the socio-demographic model (Model A) to 0.892 in the fully adjusted model (Model D). Similarly, Nagelkerke’s R^2^ increased across successive models, indicating improved explanatory power after the inclusion of vaccination status, vaccination attitudes, and trust-related factors. The inclusion of vaccination status (Model B) resulted in only a modest improvement in model performance, suggesting that vaccination status contributed additional explanatory information beyond socio-demographic and contextual characteristics but was not the sole determinant of model performance. Model performance metrics showed modest increases across successive hierarchical models. The inclusion of trust-related variables resulted in a modest increase in explanatory power despite the lack of statistically significant independent associations for individual trust-related factors. Multicollinearity diagnostics identified no evidence of problematic collinearity in the final model (Table S2, all VIF values < 2.5). Model calibration was adequate (Hosmer–Lemeshow *p* = 0.647), and discrimination was high (AUC = 0.892).

Because complete-case analysis was applied, sample sizes differed across models; therefore, comparisons of model performance should be interpreted descriptively.

### 3.9. Stratified Analyses by Healthcare Centre

To further evaluate whether the association between vaccination status and consent to medical data processing was consistent across healthcare centres, additional centre-specific logistic regression analyses were performed. A formal interaction model including healthcare centre and vaccination status could not be estimated reliably because Healthcare Centre 3 included only vaccinated participants within the analyzed study sample, resulting in complete separation. In Healthcare Centre 1, vaccinated participants were significantly more likely to provide consent than unvaccinated participants (OR = 2.59, 95% CI 1.43–4.69; *p* = 0.002). A comparable association was observed in Healthcare Centre 2 (OR = 2.04, 95% CI 1.11–3.76; *p* = 0.022), indicating that the association between vaccination status and consent was consistent across the two healthcare centres in which both vaccinated and unvaccinated participants were represented. The results of these analyses are presented in Supplementary Table S5.

## 4. Discussion

This study examined factors associated with consent to medical data processing among adult primary care patients, with particular emphasis on vaccination behaviour, vaccination-related attitudes, and trust-related factors. The most important finding was that vaccination status remained independently associated with consent across all hierarchical models. Despite this, the hierarchical analyses indicated that much of the overall predictive performance was already explained by socio-demographic characteristics and, in particular, differences in healthcare centre. Vaccination status therefore appears to represent one component of a broader pattern of engagement with healthcare rather than being the sole determinant of consent behaviour. Vaccinated participants had approximately twice the odds of providing consent to medical data processing compared with unvaccinated individuals, even after adjustment for socio-demographic characteristics, vaccination attitudes, trust-related factors, and information-seeking behaviours. This finding suggests that consent to medical data processing may reflect broader patterns of engagement with preventive healthcare. Individuals who accept recommended vaccination may be more willing to cooperate with healthcare institutions and participate in activities perceived as beneficial for public health and medical research. Previous studies have consistently shown that vaccination uptake is associated with confidence in healthcare systems, trust in healthcare professionals, and greater acceptance of preventive health interventions [13,18,21,28]. Although vaccination status should not be interpreted as a direct measure of trust [29], our findings indicate that actual preventive health behaviours may provide valuable insight into patients’ willingness to engage with healthcare-related research activities, including the secondary use of medical data.

An interesting finding was the lack of independent associations between trust-related variables and consent to medical data processing in the fully adjusted model. Participants who provided consent reported more favourable assessments of vaccination-related information and greater confidence in the healthcare system in unadjusted analyses; however, these associations did not persist after adjustment for vaccination status and other covariates. This observation suggests that trust-related factors may be reflected more strongly in actual health behaviours than in self-reported attitudes alone. Previous studies have shown that trust in healthcare institutions and healthcare professionals is associated with vaccine acceptance. However, the magnitude of these associations varies depending on how trust is conceptualized and measured [13,21,30]. Consequently, vaccination status emerged as a more robust predictor of consent than individual measures of trust or information accessibility. One possible interpretation is that vaccination behaviour may serve as a behavioural manifestation of broader engagement with healthcare systems and preventive care.

Beyond individual-level characteristics, one of the most notable findings of this study was the strong and persistent association between healthcare centre and consent to medical data processing. These differences remained substantial even after adjustment for socio-demographic characteristics, vaccination status, vaccination attitudes, and trust-related factors. The magnitude and stability of these associations suggest that contextual factors related to healthcare organizations may have contributed to differences in consent behaviour. However, because organizational characteristics were not directly measured in the present study, this interpretation should be considered hypothesis-generating rather than conclusive. These contextual factors may include differences in patient populations, organizational culture, communication practices, patient–provider relationships, continuity of care, implementation of study procedures, staff engagement, or other local characteristics that were not measured [15,16,17,31]. Future studies should investigate organizational determinants of consent behaviour in greater detail. Additional stratified analyses showed that the association between vaccination status and consent was similar in direction and magnitude across the two healthcare centres in which both vaccinated and unvaccinated participants were represented. Because Healthcare Centre 3 included only vaccinated participants in the study sample, centre-specific comparisons according to vaccination status were not possible.

The discrepancy between the univariable and multivariable analyses is also noteworthy. Participants who declined consent expressed greater vaccine-related concerns, reported lower trust in several information sources, and evaluated vaccination-related information less favourably. However, these associations were largely attenuated after adjustment for vaccination status and other variables. Similar findings have been reported in studies of vaccine hesitancy, where relationships observed in unadjusted analyses often become weaker when broader behavioural and contextual factors are considered [18,32]. These findings suggest that consent behaviour may be influenced by complex interactions between attitudes, behaviours, and healthcare experiences rather than by any single factor in isolation. This has important implications for research based on routinely collected healthcare data. Selection bias from differential consent behaviour can affect the representativeness of datasets derived from medical records. Previous studies have shown that individuals who consent to medical record processing often differ systematically from those who decline participation [4,7]. Kaushik et al. [33] highlight the complexity of vaccine hesitancy, including factors such as misinformation, a lack of trust in healthcare, cultural beliefs, and barriers to vaccine access.

By contrast, Naranjo et al. [34] indicate that patients with cancer and autoimmune diseases exhibit hesitancy due to a lack of reliable information regarding a vaccine’s impact on their current health status. As such, they require consultation with a physician or medical personnel before making a decision. The present results suggest that vaccination behaviour may represent one of the factors contributing to such differences. Consequently, researchers using consent-based medical record data should consider the possibility that study participants may not fully represent the broader patient population.

The study included a large sample of adult primary care patients recruited from healthcare centres operating in different settings. The availability of information on vaccination behaviour, vaccination attitudes, information sources, and trust-related factors enabled a comprehensive evaluation of determinants of consent to medical data processing. Furthermore, the hierarchical modelling strategy enabled an assessment of the stability of observed associations after sequential adjustment for multiple groups of covariates.

## 5. Strengths and Limitations

Several limitations of this study should be acknowledged. First, the cross-sectional design does not allow causal inferences regarding the observed associations between vaccination status, trust-related factors, healthcare centre, and consent to medical data processing. Consequently, the direction of these relationships cannot be established.

Second, the study relied on self-reported questionnaire data. Participants may have underreported vaccine-related concerns or overreported socially desirable attitudes and behaviours, potentially introducing reporting bias.

Third, consent to medical data processing was assessed using a single binary question. Consequently, the study was unable to distinguish the different motivations underlying refusal to provide consent, such as concerns about privacy, data security, distrust in healthcare institutions, lack of interest in research participation, and other personal considerations. Future studies should incorporate more detailed measures of consent-related attitudes to further understanding of the mechanisms underlying consent behaviour. In addition, vaccination status was based on participants’ self-reported questionnaire responses and operationalized as a binary variable (vaccinated/unvaccinated). Although this classification reflected the predefined study definition, it may not fully capture the diversity of individual vaccination behaviours. Consequently, some degree of exposure misclassification cannot be excluded.

Fourth, although the questionnaire was informed by established theoretical frameworks describing vaccination behaviour and trust-related determinants, several constructs were assessed using study-specific items rather than fully validated psychometric instruments. Therefore, some dimensions of vaccine confidence and healthcare engagement may not have been assessed with the same precision as studies using standardized scales.

Fifth, because complete-case analyses were performed separately for each hierarchical model, the sets of excluded participants differed between models depending on the availability of the variables introduced at each modelling step. Consequently, direct comparisons of participants included and excluded across all models were not possible. In addition, information on the number of eligible individuals who declined participation was not systematically recorded. Therefore, selection bias cannot be excluded, as participants who agreed to take part in the study may have differed from those who declined.

An additional limitation concerns the interpretation of the healthcare-centre effect. Substantial differences in consent rates were observed between participating centres and remained significant after adjustment for socio-demographic characteristics, vaccination status, vaccination attitudes, and trust-related factors. Although healthcare centre was treated as a contextual variable reflecting differences in local healthcare environments and organizational context, the present study was not designed to identify the specific mechanisms underlying these differences. Furthermore, because Healthcare Centre 3 included only vaccinated participants within the analyzed study sample, formal interaction analyses between healthcare centre and vaccination status could not be reliably estimated. Therefore, the consistency of this association could only be assessed in the remaining two healthcare centres. Variations in local administrative procedures for obtaining consent, implementation of data protection regulations, communication practices, and staff engagement in explaining consent procedures may have contributed to the observed centre-level effects. These potential mechanisms should be explored in future research.

Finally, participants were only recruited from three primary healthcare centres in Poland, which may limit the generalizability of the findings to other healthcare settings and populations. Nevertheless, the inclusion of centres operating in different organizational and geographical contexts increased the heterogeneity of the study population and enabled the evaluation of contextual influences on consent behaviour.

Despite these limitations, this study has several important strengths. It included a large sample of adult primary care patients and simultaneously evaluated socio-demographic characteristics, vaccination behaviour, vaccination-related attitudes, information-seeking behaviours, and trust-related factors. Furthermore, the hierarchical modelling strategy enabled an examination of the stability of observed associations after sequential adjustment for multiple groups of covariates, providing a comprehensive assessment of factors associated with consent to medical data processing.

## 6. Conclusions

Consent to medical data processing was independently associated with vaccination status among adult primary care patients. Vaccinated individuals were significantly more likely to provide consent than unvaccinated participants, even after adjustment for socio-demographic characteristics, vaccination-related attitudes, and trust-related factors. The findings suggest that consent behaviour may reflect broader patterns of engagement with preventive healthcare. The observed association with healthcare centre suggests that contextual factors may contribute to willingness to participate in research involving medical records. These findings have implications for the interpretation of studies based on consent-dependent healthcare data and highlight the need for further research into organizational and behavioural determinants of consent. Substantial differences in consent rates were observed between the participating healthcare centres. Although identical study procedures were implemented across participating centres, differences in patient populations, local implementation of study procedures, communication practices, staff engagement, and organizational context may also have contributed to the observed variability.

## Figures and Tables

**Table 1:** Vaccination status based on consent to medical data processing.

	Consent to Medical Data Processing *n* = 565 (%)	No Consent to Medical Data Processing *n* = 356 (%)	*p*-Value Pearson’s χ^2^ Test
Vaccinated adults	500 (88.5%)	246 (69.1%)	χ^2^ = 52.12 *p* < 0.001
Non-vaccinated adults	65 (11.5%)	110 (30.9%)	

**Table 2:** Socio-demographic characteristics of participants by consent status.

Item	Patient		*p*-Value Pearson’s χ^2^ Test
	Consent to Medical Data Processing *n* = 565 (%)	No Consent to Medical Data Processing *n* = 356 (%)	
Age (*n* = 921) Mean ± SD Median (min; max)	51.9 ± 16.47 53.0 (18.0; 98.0)	52.4 ± 16.38 52.0 (18.0; 94.0)	Mann-Whitney *p* = 0.68
Sex (*n* = 920)			
Female	344 (60.9%)	199 (56.1%)	χ^2^ = 2.1
Male	221 (39.1%)	156 (43.9%)	*p* = 0.15
Healthcare centre (*n* = 921)			
Healthcare Centre 1	247 (43.7%)	63 (17.7%)	
Healthcare Centre 2	87 (15.4%)	285 (80.1%)	χ^2^ = 395.62
Healthcare Centre 3	231 (40.9%)	8 (2.25%)	*p* < 0.001
Place of residence (*n* = 921)			
Village	269 (47.6%)	112 (31.5%)	
City < 50 k inhabitants	66 (11.7%)	164 (46.1%)	χ^2^ = 168.75
City 50–200 k inhabitants	10 (1.8%)	23 (6.5%)	*p* < 0.001
City > 200 k inhabitants	220 (38.9%)	57 (16.0%)	

**Table 3:** Vaccination-related attitudes and trust according to consent status.

Item	Patient		*p*-Value Pearson’s χ^2^ Test
	Consent to Medical Data Processing *n* = 565 (%)	No Consent to Medical Data Processing *n* = 356 (%)	
How do you assess the availability of vaccination information? (*n* = 920)			
I have no opinion	26 (4.6%)	39 (11.0%)	χ^2^ = 34.99 *p* < 0.001
Very weak	12 (2.1%)	13 (3.7%)	
Weak	41 (7.3%)	32 (9.0%)	
Medium	222 (39.4%)	91 (25.6%)	
Good	234 (41.5%)	145 (40.7%)	
Very Good	29 (5.1%)	36 (10.1%)	
How do you rate the quality of information on vaccinations? (*n* = 921)			
I have no opinion	24 (4.2%)	35 (9.8%)	χ^2^ = 43.06 *p* < 0.001
Very weak	17 (3.0%)	8 (2.2%)	
Weak	41 (7.3%)	41 (11.5%)	
Medium	227 (40.2%)	90 (25.3%)	
Good	231 (40.9%)	143 (40.2%)	
Very Good	25 (4.4%)	39 (11.0%)	
How do you assess confidence in the healthcare system? (*n* = 921)			
I have no opinion	7 (1.2%)	22 (6.2%)	χ^2^ = 27.17 *p* < 0.001
Very weak	25 (4.4%)	15 (4.2%)	
Weak	43 (7.6%)	31 (8.7%)	
Medium	322 (57.0%)	173 (48.6%)	
Good	150 (26.5%)	90 (25.3%)	
Very Good	18 (3.2%)	25 (7.0%)	

**Table 4:** Source of information based on consent to medical data processing.

Where Did You Look for Information About Vaccination? (*n* = 902)	Patient		*p*-Value Pearson’s χ^2^ Test
	Consent to Medical Data Processing *n* = 565 (%)	No Consent to Medical Data Processing *n* = 356 (%)	
Doctors			
No	108 (19.2%)	119 (35.1%)	χ^2^ = 28.48 *p* = 0.001
Yes	455 (80.8%)	220 (64.9%)	
Family and friends			
No	309 (54.9%)	226 (66.7%)	χ^2^ = 12.17 *p* < 0.001
Yes	254 (45.1%)	113 (33.3%)	

**Table 5:** Vaccination decisions, policy attitudes and views on mandatory vaccination by consent to medical data processing.

Item	Patient		*p*-Value Pearson’s χ^2^ Test
	Consent to Medical Data Processing *n* = 565 (%)	No Consent to Medical Data Processing *n* = 356 (%)	
Was the decision to vaccinate influenced to any extent by others (family, friends, social groups)? (*n* = 914)			
Never	64 (11.4%)	107 (30.3%)	χ^2^ = 66.53 *p* < 0.001
Rarely	119 (21.2%)	88 (24.9%)	
Sometimes	256 (45.6%)	107 (30.3%)	
Often	116 (20.7%)	43 (12.2%)	
Always	6 (1.1%)	8 (2.3%)	
How do you assess the vaccination policy in the country? (*n* = 914)			
Very bad	9 (1.6%)	8 (2.3%)	χ^2^ = 13.54 *p* = 0.009
Bad	42 (7.5%)	40 (11.3%)	
On average	262 (46.8%)	189 (53.4%)	
Good	233 (41.6%)	107 (30.2%)	
Very good	14 (2.5%)	10 (2.8%)	
Should vaccinations be mandatory? (*n* = 916)			
No	96 (17.1%)	87 (24.5%)	χ^2^ = 32.28 *p* < 0.001
Yes	343 (61.1%)	149 (42.0%)	
I have no opinion	122 (21.7%)	119 (33.5%)	

**Table 6:** Hierarchical logistic regression models predicting consent to medical data processing.

Variable	Model A	Model B	Model C	Model D
Age (per year)	1.02 (1.01–1.03); *p* = 0.002	1.02 (1.00–1.03); *p* = 0.011	1.02 (1.01–1.03); *p* = 0.003	1.02 (1.01–1.03); *p* = 0.004
Female sex vs. male	1.22 (0.85–1.75); *p* = 0.276	1.23 (0.85–1.76); *p* = 0.267	1.20 (0.83–1.73); *p* = 0.343	1.21 (0.81–1.80); *p* = 0.352
Healthcare Centre 2 vs. 1	0.07 (0.04–0.10); *p* < 0.001	0.06 (0.04–0.10); *p* < 0.001	0.06 (0.04–0.09); *p* < 0.001	0.06 (0.04–0.09); *p* < 0.001
Healthcare Centre 3 vs. 1	7.05 (3.30–15.08); *p* < 0.001	5.72 (2.64–12.41); *p* < 0.001	5.68 (2.58–12.53); *p* < 0.001	7.24 (2.95–17.76); *p* < 0.001
Vaccinated vs unvaccinated	—	2.07 (1.33–3.21); *p* = 0.001	2.00 (1.23–3.26); *p* = 0.005	2.27 (1.32–3.92); *p* = 0.003
Vaccine concerns index	—	—	1.08 (0.89–1.32); *p* = 0.449 *	1.08 (0.87–1.33); *p* = 0.509
Influence of others on the vaccination decision	—	—	1.00 (0.83–1.19); *p* = 0.978	0.93 (0.76–1.14); *p* = 0.508
Evaluation of vaccination policy	—	—	1.04 (0.80–1.35); *p* = 0.774	1.08 (0.80–1.46); *p* = 0.627
Mandatory vaccination: no opinion vs. no	—	—	0.81 (0.48–1.37); *p* = 0.434	0.92 (0.52–1.64); *p* = 0.784
Mandatory vaccination: yes vs no	—	—	0.99 (0.59–1.67); *p* = 0.985	1.09 (0.61–1.93); *p* = 0.775
Trust in healthcare system	—	—	—	0.77 (0.58–1.02); *p* = 0.072
Perceived accessibility of vaccination information	—	—	—	1.01 (0.77–1.34); *p* = 0.916
Physicians as information source	—	—	—	1.03 (0.65–1.63); *p* = 0.889
Family/friends as information source	—	—	—	0.99 (0.65–1.51); *p* = 0.957

* OR (95% CI) and *p*-value; reference categories: male sex, Healthcare Centre 1, unvaccinated status, mandatory vaccination = no.

**Table 7:** Goodness-of-fit and predictive performance of hierarchical logistic regression models predicting consent to medical data processing.

Model	*N*	Variables Added	AUC	Nagelkerke *R*^2^	Hosmer– Lemeshow χ^2^	*p*
A	920	Socio-demographics	0.872	0.528	12.74	0.121
B	920	+Vaccination status	0.879	0.538	10.04	0.262
C	896	+Vaccination attitudes	0.883	0.544	11.08	0.197
D	814	+Trust-related variables	0.892	0.557	6.00	0.647

## Data Availability

The data presented in this study are available from the corresponding author upon request. The data are not publicly available due to privacy protection and anonymity of patients.

## Supplementary Materials

The following supporting information can be downloaded at https://www.mdpi.com/article/10.3390/healthcare14162505/s1. Table S1. Questionnaire item used to assess consent to medical data processing, Table S2. Variance inflation factors (VIFs) for variables included in the final logistic regression model, Table S3. Level of trust in information sources, Table S4. Vaccine-related concerns by consent to medical data processing, Table S5. Stratified logistic regression analyses of the association between vaccination status and consent to medical data processing across healthcare centres.
