# Supplementary material for: Consent to Medical Data Processing Among Adult Primary Care Patients: Associations with Vaccination Behaviour and Healthcare Trust
Source: Healthcare (Basel). 2026 Aug 12;14(16):2505. doi: 10.3390/healthcare14162505 (PMC13512867; doi:10.3390/healthcare14162505)
Supplement: Supplementary file 1 [file healthcare-14-02505-s001.zip › healthcare-4438823-supplementary.pdf]

Supplementary Tables:

Table S1. Questionnaire item used to assess consent to medical data processing

Original Polish wording: *"Czy wyraża Pan/Pani zgodę na przekazanie swoich danych medycznych dotyczących historii szczepień?"*

English translation: *"Do you consent to the transfer of your medical data regarding your vaccination history?*

Table S2. Variance inflation factors (VIFs) for variables included in the final logistic regression model.

**Predictor**	**VIF**
Mandatory vaccination: yes vs no	2.22
Mandatory vaccination: no opinion vs no	1.86
Healthcare Centre 3 vs 1	1.55
Trust in healthcare system	1.53
Perceived accessibility of vaccination information	1.52
Healthcare Centre 2 vs 1	1.41
Vaccinated vs unvaccinated	1.37
Evaluation of vaccination policy	1.33
Vaccine concerns index	1.24
Influence of others	1.24
Physicians as information source	1.15
Family/friends as information source	1.11
Age (per year)	1.08
Female sex vs male	1.03

Table S3. Level of trust in information sources

How much do you trust the following sources of information about vaccines?	Patient		**p-value Pearson's χ^2 test**
	**Consent to the use of medical data (n=565)**	**No consent to the use of medical data (n=356)**	
**Internet (websites, forums, social media) (n=909)**			
Strongly disagree	34 (6.1%)	32 (9.1%)	χ^2=8.48 *p*=0.08
I don't agree	99 (17.7%)	59 (16.8%)	
Neutral	295 (52.9%)	170 (48.4%)	
I agree	123 (22.0%)	78 (22.2%)	
Strongly agree	7 (1.3%)	12 (3.4%)	
**Internet (forums, social media) (n=897)**			
Strongly disagree	27 (4.9%)	37 (10.6%)	χ^2=28.74 *p*<0.001
I don't agree	62 (11.3%)	63 (18.1%)	
Neutral	228 (41.6%)	151 (43.3%)	
I agree	190 (34.7%)	84 (24.1%)	
Strongly agree	41 (7.5%)	14 (4.0%)	
**Doctors (n=903)**			
Strongly disagree	20 (3.6%)	7 (2.0%)	χ^2=33.37 *p*<0.001
I don't agree	58 (10.5%)	20 (5.7%)	
Neutral	175 (31.6%)	102 (29.2%)	

I agree	271 (48.9%)	164 (47.0%)	
Strongly agree	30 (5.4%)	56 (16.0%)	
**Family and friends (n=902)**			
Strongly disagree	7 (1.3%)	4 (1.1%)	
I don't agree	24 (4.3%)	24 (6.9%)	
Neutral	237 (42.9%)	158 (45.3%)	χ^2=4.36 *p*=0.36
I agree	251 (45.4%)	147 (42.1%)	
Strongly agree	34 (6.1%)	16 (4.6%)	
**Media (TV, radio, press) (n=896)**			
Strongly disagree	16 (2.9%)	17 (4.9%)	
I don't agree	60 (10.9%)	49 (14.1%)	
Neutral	300 (54.6%)	189 (54.5%)	χ^2=6.63 *p*=0.16
I agree	141 (25.7%)	79 (22.8%)	
Strongly agree	32 (5.8%)	13 (3.7%)	
**Books and scientific publications (n=897)**			
Strongly disagree	23 (4.2%)	13 (3.7%)	
I don't agree	68 (12.4%)	39 (11.2%)	
Neutral	306 (55.8%)	165 (47.3%)	χ^2=11.48 *p*=0.02
I agree	138 (25.2%)	116 (33.2%)	
Strongly agree	13 (2.4%)	16 (4.6%)	
**Government organization (n=899)**			
Strongly disagree	38 (6.9%)	38 (10.9%)	
I don't agree	119 (21.6%)	72 (20.6%)	
Neutral	337 (61.3%)	183 (52.4%)	χ^2=16.85 *p*=0.002
I agree	45 (8.2%)	52 (14.9%)	
Strongly agree	11 (2.0%)	4 (1.1%)	
**Non-government organization (n=898)**			
Strongly disagree	53 (9.6%)	41 (11.8%)	
I don't agree	144 (26.1%)	62 (17.9%)	
Neutral	299 (54.3%)	200 (57.6%)	χ^2=9.18 *p*=0.06
I agree	47 (8.5%)	38 (11.0%)	
Strongly agree	8 (1.5%)	6 (1.7%)	

Table S4. Vaccine-related concerns by consent to medical data processing

What are your main concerns about vaccines?	Patient		***p*-value Pearson's χ^2 test**
	**Consent to the use of medical data (n=565)**	**No consent to the use of medical data (n=356)**	
**Possibility of complications or AEFI (n=780)**			
Strongly disagree	7 (1.4%)	9 (3.3%)	
I don't agree	29 (5.7%)	18 (6.7%)	
Neutral	162 (31.7%)	92 (34.2%)	χ^2=34.01 *p*<0.001
I agree	274 (53.6%)	99 (36.8%)	
Strongly agree	39 (7.6%)	51 (19.0%)	
**Long-term health effects**			
Strongly disagree	11 (2.2%)	7 (2.8%)	χ^2=17.38 *p*=0.001
I don't agree	38 (7.5%)	24 (9.5%)	

Neutral	165 (33.7%)	97 (38.3%)	
I agree	246 (48.7%)	86 (34.0%)	
Strongly agree	45 (8.9%)	39 (15.4%)	
**Vaccine composition (e.g. preservatives, adjuvants) (n=755)**			
Strongly disagree	14 (2.8%)	10 (4.0%)	
I don't agree	77 (15.3%)	31 (12.3%)	
Neutral	262 (52.2%)	103 (40.7%)	χ^2=25.35 *p*<0.001
I agree	124 (24.7%)	73 (28.9%)	
Strongly agree	25 (5.0%)	36 (14.2%)	
**Negative impact on the immune system (n=740)**			
Strongly disagree	11 (2.2%)	10 (4.2%)	
I don't agree	121 (24.2%)	23 (9.6%)	
Neutral	210 (42.0%)	107 (44.6%)	χ^2=51.845 *p*<0.001
I agree	127 (25.4%)	64 (26.7%)	
Strongly agree	31 (6.2%)	36 (15.0%)	
**Vaccinations are a pretext for controlling and/or microchipping the population (n=737)**			
Strongly disagree	115 (23.1%)	43 (18.0%)	
I don't agree	146 (29.3%)	45 (18.8%)	
Neutral	190 (38.2%)	86 (36.0%)	χ^2=48.47 *p*<0.001
I agree	36 (7.2%)	36 (15.1%)	
Strongly agree	11 (2.2%)	29 (12.1%)	
**Vaccinations are driven by pharmaceutical company profits, not public interest (n=748)**			
Strongly disagree	42 (8.3%)	13 (5.4%)	
I don't agree	110 (21.7%)	27 (11.3%)	
Neutral	230 (45.3%)	113 (47.1%)	χ^2=23.05 *p*<0.001
I agree	87 (17.1%)	49 (20.4%)	
Strongly agree	39 (7.7%)	38 (15.8%)	

Table S5. Stratified logistic regression analyses of the association between vaccination status and consent to medical data processing across healthcare centres.

Healthcare Centre	OR	95% CI	p
Healthcare Centre 1	2.59	1.43-4.69	0.002
Healthcare Centre 2	2.04	1.11-3.76	0.022
